# Development of a psychoeducational intervention for people affected by pancreatic cancer

**DOI:** 10.1186/s40814-019-0466-x

**Published:** 2019-06-20

**Authors:** Eryn Tong, Chris Lo, Shari Moura, Kelly Antes, Sarah Buchanan, Venissa Kamtapersaud, Gerald M. Devins, Camilla Zimmermann, Steven Gallinger, Gary Rodin

**Affiliations:** 1 Department of Supportive Care, Princess Margaret Cancer Centre, University Health Network, 700 Bay St., Suite 2303, Toronto, Ontario M5G 1Z6 Canada; 2 Institute of Medical Science, University of Toronto, Toronto, Canada; 3 Institute for Life Course and Aging, Factor-Inwentash Faculty of Social Work, University of Toronto, Toronto, Canada; 4 Department of Medicine, University of Toronto, Toronto, Canada; 5 Department of Psychiatry, University of Toronto, Toronto, Canada; 6 Department of Psychology, University of Guelph-Humber, Toronto, Canada; 7 Social and Behavioural Sciences Division, Dalla Lana School of Public Health, University of Toronto, Toronto, Canada; 8 Wallace McCain Centre for Pancreatic Cancer, Princess Margaret Cancer Centre, University Health Network, Toronto, Canada; 9 The Global Institute of Psychosocial, Palliative and End-of-Life Care, University of Toronto and Princess Margaret Cancer Centre, Toronto, Canada; 10 Department of Surgery, Toronto General Hospital, University Health Network, Toronto, Canada

**Keywords:** Pancreatic cancer, Psychoeducation, Supportive care, Palliative care, Implementation science, Intervention development

## Abstract

**Background:**

Pancreatic cancer has one of the highest mortality rates of any malignancy, placing a substantial burden on patients and families with high unmet informational and supportive care needs. Nevertheless, access to psychosocial and palliative care services for the individuals affected is limited. There is a need for standardized approaches to facilitate adjustment and to improve knowledge about the disease and its anticipated impact. In this intervention-development paper guided by implementation science principles, we report the rationale, methods, and processes employed in developing an interdisciplinary group psychoeducational intervention for people affected by pancreatic cancer. The acceptability and feasibility of implementation will be evaluated as a part of a subsequent feasibility study.

**Methods:**

The Schofield and Chambers framework for designing sustainable self-management interventions in cancer care informed the development of the intervention content and format. The Consolidated Framework for Implementation Research served as an overarching guide of the implementation process, including the development phase and the formative evaluation plan of implementation.

**Results:**

A representative team of stakeholders collaboratively developed and tailored the intervention content and format with attention to the principles of implementation science, including available resourcing. The final intervention prototype was designed as a single group-session led by an interdisciplinary clinical team with expertise in caring for patients with pancreatic cancer and their families and in addressing nutrition guidelines, disease and symptom management, communication with family and health care providers, family impact of cancer, preparing for the future, and palliative and supportive care services.

**Conclusions:**

The present paper describes the development of a group psychoeducational intervention to address the informational and supportive care needs of people affected by pancreatic cancer. Consideration of implementation science during intervention development efforts can optimize uptake and sustainability in the clinical setting. Our approach may be utilized as a framework for the design and implementation of similar initiatives to support people affected by diseases with limited prognoses.

**Electronic supplementary material:**

The online version of this article (10.1186/s40814-019-0466-x) contains supplementary material, which is available to authorized users.

## Background

Pancreatic cancer is one of the most aggressive malignancies, with an overall 5-year survival rate of only 8%, and is the fourth leading cause of cancer-related death in North America [1, 2]. It is most often diagnosed at an advanced and incurable stage since early symptoms are largely absent [1]. The threat of impending mortality can be highly distressing and patients affected by this disease demonstrate higher rates of anxiety and depression than those with other types of cancers [3, 4]. Family members show similar or even greater levels of distress than their patient counterparts [5].

Those affected by pancreatic cancer have high informational and supportive care needs regarding symptom management, communication with health care providers (HCPs), worry about loved ones, and uncertainty about the future [6]. These needs are often unmet, despite clinical practice guidelines calling for psychosocial and educational support and for early palliative care [6–8]. This is consistent with evidence that the majority of patients with advanced cancer, including those with clinically significant psychological distress, are not referred for specialized psychosocial and palliative care [9–12]. This gap in health care may be related to stigma and misunderstanding about the potential benefit of psychosocial and palliative care services [13, 14], or limited accessibility and availability [15].

Psychoeducation refers to a treatment modality that provides information for self-management within a supportive social context and embeds both education and psychological care into routine care [16–18]. Systematic reviews of studies with mixed cancer populations have shown that there are significant and sustained benefits of psychoeducational interventions in relation to emotional distress and quality of life [19–21]. Psychoeducation has often been conceptualized as an intervention for patients with earlier stage cancer, but may be no less important for those with advanced disease [8]. Multidisciplinary psychoeducation programs may be well-suited to address the early information and support needs for people affected by pancreatic cancer, yet to date, there are no targeted psychoeducational interventions for this population.

We describe here the process of developing a psychoeducational intervention to address the informational and supportive care needs of people affected by pancreatic cancer, including patients and their loved ones, following an implementation science approach. Implementation science is an emerging field that examines the processes by which interventions can be tailored and optimized for specific clinical contexts [22]. The present paper details the development process, which is an earlier stage of activity prior to the conduct of a study of feasibility. By describing this process and how it is influenced by practical and contextual factors, we hope to provide guidance for scientists and clinicians seeking to implement similar initiatives in their settings.

## Methods

We used the Schofield and Chambers [23] framework to inform the development of our intervention’s content and format. This framework seeks to promote effective and sustainable self-management interventions in cancer care. It emphasizes the targeting of interventions to cancer type and stage and tailoring them to individual needs. It also prioritizes evidence-based content, low-intensity delivery, and stakeholder acceptability.

We used the Consolidated Framework for Implementation Research (CFIR) as an overarching guide of the whole implementation process [24]. The CFIR attends to five main domains: (I) intervention characteristics (e.g.*,* evidence strength, and intervention quality and complexity); (II) the outer setting (i.e., external factors that may affect implementation, including the wider state of knowledge and policy climate); (III) the inner setting (i.e., internal organizational factors associated with readiness to implement); (IV) individual characteristics (e.g.*,* personal attributes of stakeholders, beliefs about intervention, self-efficacy); and (V) the process of implementation itself, which includes planning and forethought, engaging champions, executing the plan, and evaluating the success of the intervention and implementation. CFIR encourages formative evaluation, which is “a rigorous assessment process designed to identify potential and actual influences on the progress and effectiveness of implementation efforts” [25]. Such evaluation allows continuous quality improvement in intervention content and delivery, spanning across the phases of development and implementation.

This report conforms to the Template for Intervention Description and Replication (TIDieR) checklist, which was developed to improve the completeness of reporting and replicability of interventions [26] [see Additional file 1].

## Results

### Stepwise development of the intervention

#### Evaluating the outer and inner setting

There has been global recognition of the importance of integrated supportive and palliative care throughout the illness trajectory from diagnosis to the end of life, as reflected in recent clinical practice guidelines [8, 27, 28]. Despite such recommendations and clear clinical need, available support services are often minimal for patients with pancreatic cancer and their families [6].

The Wallace McCain Centre for Pancreatic Cancer (WMCPC) was established in 2013 at the Princess Margaret Cancer Centre (PM) in Toronto, Canada to advance the quality of care provided for this population and to develop new and innovative ways to improve outcomes and reduce the burden of disease. The WMCPC provided a unique opportunity to develop an improved model for the delivery of psychosocial care as part of usual oncology care. This center offers a comprehensive interprofessional and multidisciplinary clinic that promotes early referral to specialized psychosocial and palliative care services. Although formulating a comprehensive treatment plan is important to patients and their families, an early psychoeducational intervention in this context could also be of value to provide information and support and to promote the use of such evidence-based specialized services.

#### Involving stakeholders

The success of the development and implementation process of an intervention depends on the early involvement of key stakeholders [23, 24]. This ensures clinical relevance and commitment, and engages champions within the organization to take leadership and responsibility for sustainability. We therefore recruited an interdisciplinary team from the pancreatic oncology and supportive care clinics at our comprehensive cancer center to develop the intervention. The content developers included representatives from nursing, an oncology clinical nurse specialist (CNS) (*n* = 1, SM), social work (*n* = 2, KA, AH), dietetics (*n* = 1, SB), and psychology (*n* = 1, CL). An expert from patient education (*n* = 1, LL) ensured that the language and presentation of information were appropriate for individuals with different educational backgrounds. Implementation support was provided by research administration (*n* = 5, ET, AR, AD, SC, AF) and clinical administration (*n* = 1, VK). Conceptual oversight was provided by representatives from psychology (*n* = 1, GMD), psychiatry (*n* = 1, GR), palliative care (*n* = 1, CZ), and oncology (*n* = 1, SG). These stakeholders were involved from the time of project conception and participated in group and individual meetings to develop the intervention from September 2016 to September 2017.

#### Assessing available resourcing

The CNS, social worker, and dietitian from our team agreed to deliver the intervention jointly, with each taking primary responsibility for his or her area of expertise. As part of usual care, these professionals had been providing individualized assessments and care regarding pain and symptoms, nutrition, advance care planning, and how to live well with pancreatic cancer. However, they recognized the greater efficiency of a group format and the potential value of working together to deliver the psychoeducational intervention [19]. A group intervention format was considered to be the most clinically feasible and cost- and time-efficient to provide information to patients and families. Group psychoeducational interventions can also help normalize circumstances of disease and reduce uncertainties of the future, by talking to others in a similar situation [29], and have been used to support both patients and caregivers affected by cancer [19, 30, 31]. The ongoing role of the team members within the WMCPC would also allow the intervention to be sustained subsequently as part of routine care. Early consultation with these professionals suggested compatibility between their perceived clinical roles and the goals of the intervention. As we continued to develop the intervention and to conduct practice sessions, the team became increasingly more invested in and felt shared ownership of this implementation effort. Such strengthening of interpersonal ties has been found to be necessary for sustainable implementation [32].

There was debate during the development process about the intervention “dose,” or the number of sessions needed for optimal clinical benefit. The degree of benefit people obtain from psychosocial interventions is typically associated with the number of sessions they receive [20], but this also may increase the costs and burden of delivery and participation. For this reason, low-intensity designs are increasingly adopted in stepped-care models of psychological care, to provide services efficiently that respond to need, to improve access and maximize cost-effectiveness [23, 33]. This includes brief single session psychoeducational interventions, which have shown benefits in relation to knowledge, preparedness, and unmet needs, and may have greater potential for sustainability [30, 31]. Balancing these factors, we created an intervention prototype consisting of a single session lasting 1.5 h. The first hour focused on delivering content; the last half hour was reserved for questions. We offered the intervention on a rotating, biweekly basis to accommodate space and time constraints. This low-intensity model could be integrated easily into the flow of usual care.

#### Establishing the evidence base for the content of the intervention

Considerable evidence demonstrates that patients with advanced cancers experience a range of physical and psychosocial challenges [7, 34]. In pancreatic cancer, these include: (1) problems with digestion and diet, poor appetite, and rapid weight loss [35]; (2) physical symptoms such as abdominal and back pain, nausea, jaundice, and diarrhea [36, 37]; (3) fears and concerns about the future [38]; and (4) adaptation to the impact of progressive disease on self and close others [39–41]. The encouragement of open communication and partnership with the health care team early in the disease trajectory improves symptom management and end-of-life outcomes, and can facilitate timely and appropriate referral and acceptance of specialized psychosocial and palliative care services [42–44].

The experience of cancer affects not only patients, but also their intimate others [45]. Family members fulfill many important caregiving duties for their loved ones affected by a cancer diagnosis, yet their roles and unique supportive care needs are often underestimated. Without adequate support, the burden of caregiving and worry about losing a loved one can lead to poor health and distress [5, 46, 47], especially as the disease progresses [48]. Supportive interventions that treat patients and their families as a single group implicitly acknowledge interdependencies among members of the family system [49], which is consistent with the principles of palliative care [27].

#### Tailoring the intervention

We designed the intervention to welcome all interested loved ones to attend with the patient. It was designed to be easily comprehensible to a wide audience without overwhelming participants with detail. The script was phrased in plain language with a Flesch Reading Ease (FRE) score of 65.1% [50] and Flesch-Kincaid reading grade level of 8.8 [51], indicating an eighth-grade reading level. The group format and circular seating arrangement of the group were chosen to encourage interactive discussion among attendees and facilitators, allowing for interjections and requests for clarification throughout the session. We included print handouts for note-taking to reduce the burden of recall. We focused on building a sense of trust and rapport with the health care team and offered to meet for individualized consults post-session.

Our interdisciplinary team of stakeholders reviewed the literature to generate an initial list of topics for the intervention. Upon further team consultation and refinement, we arrived at our key content areas. They were chosen to comprehensively address the range of concerns as identified in the literature and from clinical experience (see Table 1 for key content areas and main discussion points). These areas were related to the physical effects of pancreatic cancer, its impact on psychological and social wellbeing, ways to address those impacts (e.g., communication and planning for the end of life), and resources that can provide additional support. Generation of key content areas was followed by the development of a treatment manual for *Living Well with Pancreatic Cancer* [see Additional file 2]. Participants received a folder that included printed slides, informational pamphlets, and details about hospital network- and community-based programs that provide relevant support. The first author (ET) and education specialist (LL) assembled the PowerPoint presentation and developed the accompanying script to ensure the quality of content and design. In particular, they emphasized the use of plain language, readability, and developed an appropriate layout that included both text and graphic content.

**Table 1:** Key content areas and discussion points of the psychoeducational intervention

Key content areas	Discussion points
1. Disease management	• Describe nutrition goals to maintain physical function and quality of life, and when a dietitian consultation may be required
	• Discuss how to manage common symptoms related to pancreatic cancer, promote partnership with the health care team for symptom management, and clarify the role and goals of palliative care services
2. Personal and family impact of cancer	• Discuss the impact of cancer on personal and family emotions and relationships, and the importance of maintaining a balance between hopes and fears, and continuing to live life meaningfully
3. Communication with loved ones and health care providers	• Emphasize the importance of open communication with loved ones and health care providers throughout discussions of other key content areas
4. Planning for the future	• Explain the importance of advance care planning
5. Supportive care services	• Describe the available supportive care services offered within the hospital and in the community for the patient and family

The delivery of the intervention was tailored to broach difficult topics in a supportive and non-threatening way. Our team acknowledged the urgency and perceived threat of discussions of advance care planning and palliative care for this population. Therefore, the order in which topics were introduced was organized to commence with material that was more practical and then to proceed to more future-oriented topics associated with living with advanced cancer. The intervention first addressed practical issues involving nutrition and self-management of symptoms, including tips on how to eat and maintain weight during treatment, pain management, bowel movements, and how to cope with disease and treatment-induced nausea using both dietary and medication monitoring. This was followed by information about palliative care and advance care planning. To dispel myths surrounding the term palliative care, we defined it as focusing on improving the quality of life in patients and families, and including pain and symptom management for individuals at any age or point in the illness trajectory, regardless of the course of treatment, aligning with recent clinical recommendations [14, 52]. We explained that adapting to advanced disease requires engaging in and living life meaningfully, while simultaneously planning and preparing for all eventualities, including death. This challenge was described as similar to following two, divergent paths at the same time. This analogy of a “double road” or “double awareness” has been found to be clinically useful [53–55].

The last issues to be discussed were the impact of cancer on patients and their families, and available supportive-care services, including hospital-based services (e.g., social workers, psychologists, psychiatrists, spiritual care workers, mindfulness-based cognitive therapy, psychotherapy tailored for advanced cancer [56], and community-based services (e.g., Gilda’s Club, Wellspring, Canadian Cancer Society, Pancreatic Cancer Canada, Craig’s Cause Pancreatic Cancer Society). We emphasized that patients and families face illness together, and discussed both the importance and difficulty of sustaining open communication about physical, emotional, and existential concerns when they arise. Attendees were encouraged to seek and accept help from others. Throughout the session, we sought to engage patients and family in honest, supportive dialog as a demonstration of the value of professional support [57].

#### Planning for a formative evaluation

To assess the success of our implementation effort and to provide strategic information that may guide its further improvement, the intervention described in this paper is currently being tested in a feasibility study using a mixed-methods approach. Outcomes include the rate of referral to the intervention and number of patients and loved ones who attend; interview feedback from attendees about the timing, acceptability, and value of the intervention, and their suggestions for its improvement; and feedback from health care providers in the clinic about the process and feasibility of intervention implementation. This study will aim to characterize the feasibility and acceptability of the intervention and its implementation process in an ambulatory pancreatic oncology clinic at a large tertiary cancer center.

### Summary

We report here on the stepwise development of an interdisciplinary-led, group psychoeducational intervention to meet the informational and supportive care needs of individuals affected by pancreatic cancer. *Living Well with Pancreatic Cancer* was developed by embedded health care professionals and based on their clinical experiences, the research literature, and implementation considerations. We hope that this paper may aid potential replication efforts and be helpful to other researchers seeking similar endeavors. We look forward to reporting the feasibility results when they become available.

Living with pancreatic cancer is highly challenging for both patients and their loved ones. It may epitomize the general public’s worst fears about having cancer because of its sudden onset, limited treatment options and effectiveness, and rapid course of deterioration. Given the current poor rate of survival for most patients with this disease, there is an urgent need to continue to promote adaptation and preparation and to provide support for patients as soon as possible after diagnosis. *Living Well with Pancreatic Cancer* is consistent with guidelines to provide early, dedicated palliative, and supportive care concurrently with oncology care to improve the overall standard of care [8]. Its implementation into routine practice disseminates knowledge and promotes reflection about the foreseeable physical and psychosocial concerns that arise over the course of this illness. Psychoeducation may constitute the first line of supportive intervention, with more specialized individual treatment provided subsequently within a stepped-care framework or tiered model of supportive care delivery [58].

## Conclusion

The present study describes the development of an interdisciplinary-led intervention to support patients and caregivers following a recent diagnosis of pancreatic cancer. We considered implementation science principles during intervention development, to promote future uptake and sustainability in the clinical setting. This approach can be used to inform the design and implementation of similar initiatives to support people affected by other diseases with limited prognoses.

## Additional files

Additional file 1: Completed TIDieR Checklist. (PDF 57 kb)

Additional file 2: *Living Well with Pancreatic Cancer* Intervention Manual. (PDF 945 kb)

## Data Availability

Data sharing is not applicable to this article as no datasets were generated or analyzed during the current study. The intervention manual is submitted as Additional file 1 for publication.

## Abbreviations

CFIR: Consolidated Framework for Implementation Research
CNS: Clinical nurse specialist
FRE: Flesch Reading Ease
HCP: Health care provider
TIDieR: Template for Intervention Description and Replication
WMCPC: Wallace McCain Centre for Pancreatic Cancer
