# Supplementary material for: Development of a psychoeducational intervention for people affected by pancreatic cancer
Source: Pilot Feasibility Stud. 2019 Jun 20;5:80. doi: 10.1186/s40814-019-0466-x (PMC6584982; doi:10.1186/s40814-019-0466-x)
Supplement: Supplementary file 1 — Completed TIDieR Checklist. (PDF 57 kb) [file 40814_2019_466_MOESM1_ESM.pdf]

Additional File 1
Completed TIDieR Checklist

TIDieR Template for Intervention Description and Replication

The TIDieR (Template for Intervention Description and Replication) Checklist*:
Information to include when describing an intervention and location of the information

Item #	Item	Where located**
	**BRIEF NAME**	
1	Provide the name or a phrase that describes the intervention.	*Living Well with Pancreatic Cancer*: a group interdisciplinary-led psychoeducational intervention for people affected by pancreatic cancer
	**WHY**	
2	Describe any rationale, theory, or goal of the elements essential to the intervention.	The *Living Well with Pancreatic Cancer* intervention was developed using the Schofield and Chambers' framework for self-management interventions in cancer care and the Consolidated Framework for Implementation Research. It aims to address the information and supportive care needs of patients and loved ones affected by pancreatic cancer.
	**WHAT**	
3	Materials: Describe any physical or informational materials used in the intervention including those provided to participants or used in intervention delivery or in training of intervention providers. Provide information on where the materials can be accessed (e.g. online appendix, URL).	The intervention includes a single 1.5 hour psychoeducational session, with one hour of content presentation and a half hour for questions and discussion. Table 1 provides details of the key areas of intervention content. Details of the programme slides and script used by intervention providers can be found in Additional File 2. Participants were given an information folder with printed slides, informational pamphlets, details about hospital- and community based support programs (see Additional File 2 for details).

4	Procedures: Describe each of the procedures, activities, and/or processes used in the intervention, including any enabling or support activities.	All new patients seen at the pancreatic cancer outpatient clinic are informed about and encouraged to attend the intervention with their loved ones. An appointment is scheduled for interested patients and loved ones. Participants attend the session and are provided with the informational folder at the start. The slide deck are presented and interactively discussed for one hour, and the last half hour is dedicated to further questions and discussions.
	**WHO PROVIDED**	
5	For each category of intervention provider (e.g. psychologist, nursing assistant), describe their expertise, background, and any specific training given.	The intervention is delivered by 3 health professionals (*i.e.,* nurse, social worker, dietitian) with experience in working with patients and loved ones affected by pancreatic cancer. Facilitators helped develop the intervention manual and slides, and co-led practice sessions prior to recruiting to the study.
	**HOW**	
6	Describe the modes of delivery (*e.g.* face-to-face or by some other mechanism, such as internet or telephone) of the intervention and whether it was provided individually or in a group.	The intervention will be delivered by an interdisciplinary team in a face-to-face group session, consisting of patients and loved ones. The room was formatted in a circular seating arrangement, with facilitators interspersed amongst participants.
	**WHERE**	
7	Describe the type(s) of location(s) where the intervention occurred, including any necessary infrastructure or relevant features.	The intervention will be delivered at a tertiary comprehensive cancer hospital, in an accessible room near the outpatient clinic.
	**WHEN and HOW MUCH**	
8	Describe the number of times the intervention was delivered and over what period of time including the number of sessions, their schedule, and their duration, intensity or dose.	The intervention will be delivered as biweekly 1.5 hour sessions. Patients and loved ones are encouraged to come after a confirmed diagnosis of pancreatic cancer has been made, but are welcome to attend at any point throughout the illness trajectory. Sessions are scheduled outside of outpatient clinic times, to account for the health care professionals' availabilities.
	**TAILORING**	

**9**	If the intervention was planned to be personalised, titrated, or adapted, then describe what, why, when, and how.	This item defines tailoring as personalizing an intervention for the individual (*e.g.,* individual titration). In this sense, *Living Well with Pancreatic Cancer* is not personalized. However, given the interactive group format, there is inherent minimal personalization involved that is dependent on the interests of the individuals in each group.
	**MODIFICATIONS**	
**10‡**	If the intervention was modified during the course of the study, describe the changes (what, why, when, and how).	N/A (intervention not yet delivered)
	**HOW WELL**	
**11**	Planned: If intervention adherance or fidelity was assessed, describe how and by whom, and if any strategies were used to maintain or improve fidelity, describe them.	N/A (intervention not yet delivered)
**12‡**	Actual: If intervention adherence or fidelity was assessed, describe the extent to which the intervention was delivered as planned.	N/A (intervention not yet delivered)

** **Authors** - use N/A if an item is not applicable for the intervention being described. **Reviewers** – use '?' if information about the element is not reported/not sufficiently reported.

† If the information is not provided in the primary paper, give details of where this information is available. This may include locations such as a published protocol or other published papers (provide citation details) or a website (provide the URL).
‡ If completing the TIDieR checklist for a protocol, these items are not relevant to the protocol and cannot be described until the study is complete.

* We strongly recommend using this checklist in conjunction with the TIDieR guide (see BMJ 2014;348:g1687) which contains an explanation and elaboration for each item.

* The focus of TIDieR is on reporting details of the intervention elements (and where relevant, comparison elements) of a study. Other elements and methodological features of studies are covered by other reporting statements and checklists and have not been duplicated as part of the TIDieR checklist. When a **randomised trial** is being reported, the TIDieR checklist should be used in conjunction with the CONSORT statement (see www.consort-statement.org) as an extension of **Item 5 of the CONSORT 2010 Statement**. When a **clinical trial protocol** is being reported, the TIDieR checklist should be used in conjunction with the SPIRIT statement as an extension of **Item 11 of the SPIRIT 2013 Statement** (see www.spirit-statement.org). For alternate study designs, TIDieR can be used in conjunction with the appropriate checklist for that study design (see www.equator-network.org).
